# Variational principles for stochastic fluid dynamics

**DOI:** 10.1098/rspa.2014.0963

**Published:** 2015-04-08

**Authors:** Darryl D. Holm

**Affiliations:** Department of Mathematics, Imperial College, London, UK

**Keywords:** geometric mechanics, stochastic fluid models, cylindrical stochastic processes, multiscale fluid dynamics, symmetry reduced variational principles

## Abstract

This paper derives stochastic partial differential equations (SPDEs) for fluid dynamics from a stochastic variational principle (SVP). The paper proceeds by taking variations in the SVP to derive stochastic Stratonovich fluid equations; writing their Itô representation; and then investigating the properties of these stochastic fluid models in comparison with each other, and with the corresponding deterministic fluid models. The circulation properties of the stochastic Stratonovich fluid equations are found to closely mimic those of the deterministic ideal fluid models. As with deterministic ideal flows, motion along the stochastic Stratonovich paths also preserves the helicity of the vortex field lines in incompressible stochastic flows. However, these Stratonovich properties are not apparent in the equivalent Itô representation, because they are disguised by the quadratic covariation drift term arising in the Stratonovich to Itô transformation. This term is a geometric generalization of the quadratic covariation drift term already found for scalar densities in Stratonovich's famous 1966 paper. The paper also derives motion equations for two examples of stochastic geophysical fluid dynamics; namely, the Euler–Boussinesq and quasi-geostropic approximations.

## 1. Introduction

In this paper, we propose an approach for including stochastic processes as cylindrical noise in systems of evolutionary partial differential equations (PDEs) that derive from variational principles which are invariant under a Lie group action. Such dynamical systems are called Euler–Poincaré equations [1,2]. The main objective of the paper is the inclusion of stochastic processes in ideal fluid dynamics, in which case the variational principle is invariant under the Lie group of smooth invertible maps acting to relabel the reference configuration of Lagrangian coordinates for the fluid. Examples include Euler's fluid equations for incompressible flows and also geophysical fluid dynamics (GFD) of ocean and atmosphere circulation. The approach is via a stochastic extension of the well-known variational derivation of the Eulerian representation of ideal fluid dynamics [2].

The resulting stochastic partial differential equations (SPDEs) contain a type of multiplicative, cylindrical, Stratonovich noise that depends on the *gradients* of the solution variables. This unfamiliar feature does not interfere with the passage to the Itô representation, though, since the space variable is treated merely as a parameter when dealing with cylindrical noise. That is, one may regard the cylindrical noise process as a finite dimensional stochastic process parametrized by ***x*** (the space variable). Then, the Stratonovich equation makes analytical sense pointwise, for each fixed ***x***. Once this is agreed, then the transformation to Itô by the standard method also makes sense pointwise in space.

To specify the spatial correlations required in applications of the cylindrical Stratonovich noise process, we advocate the strategy of applying proper orthogonal decompositions (PODs) to the appropriate numerical and observational data available at resolvable scales [3]. One may then regard the stochastic process as arising physically as the effect of sub-grid scale degrees of freedom on the resolved scales of fluid motion.^1^

In more detail, the aim of this paper is to use the methods of geometric mechanics to enable fluid dynamics to be effectively adapted to include Stratonovich stochastic processes. Towards this end, we derive a set of stochastic fluid equations for the motion of an either compressible, or incompressible fluid in $R^{3}$ from a *stochastically constrained* variational principle *δS*=0, with action, *S*, given by

1.1 $$S(u,p,q)=\int (\ell (u,q)\;dt+{\left\langle p,\;dq+{£}_{dx_{t}}q\right\rangle }_{V}),$$

where ℓ(*u*,*q*) is the unperturbed deterministic fluid Lagrangian, written as a functional of velocity vector field *u* and advected quantities *q*. Here, $u\in X(R^{3})$ is the fluid velocity vector field, the angle brackets

1.2 $${\left\langle p,q\right\rangle }_{V}:=\int \langle p(x),q(x,t)\rangle \;dx$$

denote the spatial *L*^2^ integral over the domain of flow of the pairing 〈*p*,*q*〉 between elements *q*∈*V* and their dual elements *p*∈*V* *. In (1.1), the quantity *p*∈*V* * is a Lagrange multiplier and £_*dx*_*t*__*q* is the *Lie derivative* of an advected quantity *q*∈*V* , along a vector field *dx*_*t*_ defined by the following sum of a drift velocity *u*(*x*,*t*) and Stratonovich stochastic process with *cylindrical noise* parametrized by spatial position *x*, [4,5]

1.3 $$dx_{t}(x)=u(x,t)\;dt-\sum_{i}\xi_{i}(x)\circ dW_{i}(t).$$

**Remark 1.1**

Note at the outset that *dx*_*t*_(*x*) is a stochastic vector field parametrized by the spatial position *x*. The corresponding time integral of this vector field is a stochastic *function* (*ω*,*t*,*x*)→*x*_*t*_(*ω*,*x*) defined as^2^

1.4 $$x_{t}(x)=x_{0}(x)+\int_{0}^{t}u(x,s)\;ds-\sum_{i}\int_{0}^{t}\xi_{i}(x)\circ dW_{i}(s).$$

In particular, the *x*_*t*_ treated here is *not* the stochastic flow satisfying the SDE

1.5 $$x_{t}(x)\neq x+\int_{0}^{t}u(x_{s}(x),s)\;ds-\sum_{i}\int_{0}^{t}\xi_{i}(x_{s}(x))\circ dW_{i}(s),$$

which arises in other treatments of stochastic fluid dynamics, e.g. [6]. This difference in the definitions between the stochastic vector fields treated here and the usual notation in the SDE setting for stochastic fluids is necessary for our purposes here, and it greatly facilitates the analysis. We want to combine geometric mechanics with stochastic analysis for fluids. However, the usual flow relationships between Lagrangian particle maps and Eulerian fluid velocities are problematic in the stochastic setting, since expressions involving tangent vectors to stochastic processes are meaningless. Therefore, we shall develop a theory *entirely* within the Eulerian interpretation of fluid dynamics. That is, all the variables discussed below will depend parametrically on the spatial coordinate *x*.

At this point, we have introduced Stratonovich stochasticity into the action principle for fluids in (1.1) through the constraint that advected quantities *q* should evolve by following the Stratonovich perturbed vector field. This advection law is formulated as a Lie derivative with respect to the Stratonovich stochastic vector field in (1.3). For mathematical discussions of Lie derivatives with respect to stochastic vector fields [7,8,9,10,11].

The definition of Lie derivative we shall use here is the standard Cartan definition in terms of the action of the differential operator ***d*** acting on functions of the spatial coordinate *x*, for example,

1.6 $${£}_{dx_{t}}q=\text{d}(i_{dx_{t}}q)+i_{dx_{t}}\;\text{d}q,$$

where *i*_*dx*_*t*__ ***d****q* denotes insertion of the vector field *dx*_*t*_(*x*) into the differential form ***d****q*(*x*), with the usual rules for exterior calculus. For a review of exterior calculus in the context of fluid dynamics, e.g. [12].

One may interpret *dx*_*t*_(*x*) in (1.3) as the decomposition of a vector field defined at position *x* and time *t* into a time-dependent drift velocity *u*(*x*,*t*) and a stochastic vector field. The time-independent quantities *ξ*_*i*_(*x*) with *i*=1,2,…,*K* in the cylindrical stochastic process are usually interpreted as ‘diffusivities’ of the stochastic vector field, and the choice of these quantities must somehow be specified from the physics of the problem to be considered. Here, we will specify the diffusivities *ξ*_*i*_(*x*) as a set of preassigned physical spatial correlations for the stochasticity. This information is to be provided during the formulation of the problem under consideration. As an example, we will interpret the *ξ*_*i*_(*x*) with *i*=1,2,…,*K* as spatial correlations obtained from, say, coarse-grained observations or computations (e.g. as PODs) which supply the needed information for *K* independent Wiener (Brownian) processes, *dW*_*i*_(*t*), in the Stratonovich sense. Note that the number of vector fields *K* need not be equal to the number of spatial dimensions.

The *L*^2^ pairing 〈⋅,⋅〉_*V*_ in the stochastic variational principle (SVP) written in (1.1) with Lagrange multiplier *p*∈T*V enforces the *advection condition* that the quantity *q*∈*V* is preserved along the Stratonovich stochastic path. (1.3), namely,

1.7 $$dq+{£}_{dx_{t}}q=0.$$

The advection relation (1.7) for the quantities *q*∈*V* may be regarded as a *stochastic constraint* imposed on the variational principle (1.1) via the Lagrange multiplier *p*. Requiring that the solution to (1.7) exists locally in time amounts to assuming that the ‘back-to-labels’ map for the solution of (1.3) exists locally in time for the flow generated by the vector field (1.3), cf. [13,14].

An interesting physical situation occurs when numerical and observational data are available for comparison with the dynamics of the stochastic model. This is the situation in which the standard method of PODs may become useful. In particular, one may compute the PODs corresponding to the dominant correlations in this numerical and observational data, then select among those PODs according to well-defined physical criteria the modes corresponding to *ξ*_*i*_(*x*) with *i*=1,2,…,*K* for finite *K*. In that case, the stochastic terms in equation (1.3) represent a specific finite set of spatially correlated, but unresolved, degrees of freedom represented by noise, that are coupled nonlinearly to the deterministic evolution of velocity *u* and advected quantity *q* through the Lagrange-to-Euler stochastic tangent map in equation (1.3). For example, one may choose the PODs *ξ*_*i*_(*x*) with *i*=1,2,…,*K* as the first *K* eigenfunctions of the correlation tensor for the observed or simulated velocity fields determined by [3]

1.8 $$\int \langle u(x)u(x^{\prime })\rangle \xi_{i}(x^{\prime })\;dx^{\prime }=\lambda_{i}^{2}\xi_{i}(x)\;\text{no sum on }i=1,2,\ldots ,K\;\text{with }\lambda_{1}>\lambda_{2}>\cdots >\lambda_{K},$$

so that these first *K* eigenfunctions represent the highest correlations of velocity and thus account for the greatest fraction of kinetic energy in the data, compared with any other set of the same dimension. (The eigenvalues $\lambda_{i}^{2}$ are all positive because of their interpretation as the relative kinetic energies of the eigenfunctions *ξ*_*i*_(*x*).) Naturally, one might want to weight the eigenfunctions in equation (1.3) as *ξ*_*i*_(*x*)→λ_*i*_*ξ*_*i*_(*x*) (the positive square roots λ_*i*_ of their relative kinetic energy eigenvalues $\lambda_{i}^{2}$), to give them the correct dimensional meaning (velocity) and relative importance.

**Remark 1.2**

Of course, the strategy of dividing the solution into essential and non-essential modes, then replacing the dynamical effects of the non-essential modes by noise is well known. For example, one may refer to Majda *et al.* [15] for the history and motivation for this approach, as well as descriptions of their own approach, called stochastic mode reduction (SMR). Some versions of SMR can be based, for example, on truncating the Fourier representation of the numerical method at a certain level that defines the essential modes, then stochastically modelling the nonlinear interactions among the remaining modes, deemed to be non-essential.

### (a) Main results

The SPDEs which will result from the stochastically constrained variational principle *δS*=0 for *S* defined in (1.1) are expressed in Stratonovich form in terms of the Lie-derivative operation £_*dx*_*t*__ as

1.9 $$d\frac{\delta \ell }{\delta u}+{£}_{dx_{t}}\frac{\delta \ell }{\delta u}-\frac{\delta \ell }{\delta q}⋄q\;dt=0\;\text{and}\;dq+{£}_{dx_{t}}q=0,$$

in which *dx*_*t*_ is the Eulerian vector field in equation (1.3) for the velocity along the Lagrangian Stratonovich stochastic path and the diamond operation (⋄) will be explained below in definition 1.5.

The corresponding Itô forms of these equations are

1.10 $$\begin{array}{rl} d\frac{\delta \ell }{\delta u}+{£}_{d{\hat{x}}_{t}}\frac{\delta \ell }{\delta u}-\frac{\delta \ell }{\delta q}⋄q\;dt & =\frac{1}{2}\sum_{i,j}{£}_{\xi_{j}(x)}\left({£}_{\xi_{i}(x)}\frac{\delta \ell }{\delta u}\right)[dW_{i}(t),dW_{j}(t)]\\ \;\text{and}\;dq+{£}_{d{\hat{x}}_{t}}q & =\frac{1}{2}\sum_{i,j}{£}_{\xi_{j}(x)}({£}_{\xi_{i}(x)}q)[dW_{i}(t),dW_{j}(t)], \end{array}\}$$

in which the Eulerian vector field $d{\hat{x}}_{t}$ tangent to the Lagrangian Itô stochastic path ${\hat{x}}_{t}$ is given by

1.11 $$d{\hat{x}}_{t}=u(x,t)\;dt-\sum_{i}\xi_{i}(x)\;dW_{i}(t).$$

In equation (1.10), the quantities [*dW*_*i*_(*t*),*dW*_*j*_(*t*)] with *i*,*j*=1,2,…,*K* for *K* independent stochastic processes denote the quadratic covariations of the temporal Itô noise. For Brownian processes, these quantities satisfy [*dW*_*i*_(*t*), *dW*_*j*_(*t*)]=0 unless *i*=*j* and satisfy [*dW*_*j*_(*t*), *dW*_*j*_(*t*)]=*dt* (no sum) [16]. Hereafter, in choosing Brownian processes, we may write [*dW*_*i*_(*t*), *dW*_*j*_(*t*)]=*δ*_*ij*_ *dt*.

### (b) Interpretations of the main results as Kelvin circulation theorems for incompressible flow

The interpretations of the equations (1.9) and (1.10) may be expressed quite succinctly in the case of incompressible flow. In that case, volume elements are preserved and the advected variables *q* are absent. The corresponding Kelvin circulation theorems about the evolution of the integral of the *circulation 1-form* (*δ*ℓ/*δu*) around a material loop are, as follows.

For the Stratonovich case,

1.12 $$d\oint_{c(t)}\frac{\delta \ell }{\delta u}=0\;\text{for loops }c(t)\text{ governed by}\;\;dc(t)=-{£}_{dx_{t}}c(t),$$

and equivalently for the Itô case,

1.13 $$d\oint_{\hat{c}(t)}\frac{\delta \ell }{\delta u}=\oint_{\hat{c}(t)}\frac{1}{2}\sum_{i,j}{£}_{\xi_{j}(x)}\left({£}_{\xi_{i}(x)}\frac{\delta \ell }{\delta u}\right)\delta_{ij}\;dt\;\text{for}\;d\hat{c}(t)=-{£}_{d{\hat{x}}_{t}}\hat{c}(t).$$

Thus, the Kelvin theorem in (1.12) shows that circulation is *conserved* for loops moving along the Stratonovich stochastic path with velocity vector field *dx*_*t*_ in equation (1.3). However, perhaps not surprisingly, because the velocities of the loops are different, the equivalent Kelvin theorem (1.13) shows that the Stratonovich circulation law is *masked* in the Itô formulation for loops moving along the Itô stochastic path with velocity $d{\hat{x}}_{t}$ in equation (1.11), because the Itô terms cannot be expressed as a single Lie derivative of the circulation 1-form. Specifically, the circulation created in these Itô loops in (1.13) is determined from the spatial correlation vector fields *ξ*_*i*_(*x*).

The proofs of these Kelvin circulation theorems are straightforward. For example, equation (1.13) is proved, as follows:

1.14 $$\frac{d}{dt}\oint_{\hat{c}(t)}\frac{\delta \ell }{\delta u}=\oint_{\hat{c}(t)}(\partial_{t}+{£}_{d{\hat{x}}_{t}})\frac{\delta \ell }{\delta u}=\frac{1}{2}\oint_{\hat{c}(t)}\sum_{j}{£}_{\xi_{j}(x)}\left({£}_{\xi_{j}(x)}\frac{\delta \ell }{\delta u}\right),$$

in which the last step is made by referring to equation (1.10) for the case when the advected quantities *q* are absent.

**Remark 1.3**

When the noise is completely uncorrelated in each spatial dimension so that *ξ*_*i*_=const., for *i*=1,2,3, and also provided *q*=1 (volume preservation), the double Lie-derivative operator $\sum_{i}{\text{ANAND }}_{\xi_{i}}({\text{ANAND }}_{\xi_{i}}\;\cdot )$ appearing in equations (1.10), (1.13) and (1.14) reduces to the metric Laplacian operator, Δ=div grad. Some foundational results on related SPDEs can be found in Flandoli [17,18] and references therein.

**Remark 1.4**

It remains to define the diamond operation (⋄) appearing in equations (1.9) and (1.10). The diamond operation (⋄) appears when we include potential energy terms depending on the advected variables *q* in the stochastically constrained variational principle in (1.1).

**Definition 1.5 (The diamond operation)**

On a manifold *M*, the diamond operation $(⋄):T^{*}V\to X^{*}$ is defined for a vector space *V* with (*q*,*p*)∈T*V and vector field ξ∈X is given in terms of the Lie-derivative operation £_*u*_ by

1.15 $${\left\langle p⋄q,\xi \right\rangle }_{X}:={\left\langle p,-{£}_{\xi }q\right\rangle }_{V}$$

for the pairings ${\left\langle \cdot ,\cdot \right\rangle }_{V}:T^{*}V\times TV\to R$ and ${\left\langle \cdot ,\cdot \right\rangle }_{X}:X^{*}\times X\to R$ with $p⋄q\in X^{*}$.

**Remark 1.6 (Momentum map)**

The quantity J(q,p)=p⋄q in (1.15) defines the *cotangent-lift momentum map* for the action of the vector fields ξ∈X on the vector space *V* [2]. In terms of the momentum map p⋄q, the action integral *S* in (1.1) for the SVP *δS*=0 may be written *equivalently* as

1.16 $$S(u,p,q)=\underset{\mathrm{Lebesgue}\;\mathrm{integral}}{\underbrace{\int \left(\ell (u,q)+{\left\langle p,\frac{dq}{dt}+{£}_{u}q\right\rangle }_{V}\right)dt}}+\underset{\mathrm{Stratonovich}\;\mathrm{integral}}{\underbrace{\int \sum_{i}{\left\langle p⋄q,\xi_{i}(x)\right\rangle }_{X}\circ dW_{i}(t)}}.$$

Thus, the vector-field coupling between the deterministic and stochastic parts of the SVP in this equivalent form in (1.16) of the action integral (1.1) is through the momentum map in (1.15). In finite dimensions, formula (1.16) fits within the framework of [19,20] for stochastic canonical Hamilton equations and generalizes the work of [21] for stochastic variational integrators applied to the rigid body. This sort of momentum-map coupling in finite dimensions was also noted for symmetry reduction of mechanical systems with stochastic non-holonomic constraints studied in [22].

**Remark 1.7 (Variational derivations of the Navier–Stokes equations from stochastic equations)**

The derivation of the Navier–Stokes equations in the context of stochastic processes has a long and well-known history (e.g. [13,14] and references therein). Previous specifically variational treatments of stochastic fluid equations generally started from the famous remark by Arnold [23] (about Euler's equations for the incompressible flow of an ideal fluid being geodesic for kinetic energy given by the *L*^2^ norm of fluid velocity) and they have mainly treated Itô noise in this context. For more discussion of these variational derivations of stochastic fluid equations and their relation to the Navier–Stokes equations, one should consult original sources such as Inoue & Funaki [24], Rapoport [25,26], Gomes [27], Cipriano & Cruzeiro [28], Constantin & Iyer [13], Eyink [14], Gliklikh [29] and Arnaudon *et al.* [6]. We emphasize that the goal of this work is to derive SPDEs for fluid dynamics by following the stochastic variational strategy outlined above. It is not our intention to derive the Navier–Stokes equations in the present context. However, as mentioned previously, in imposing the stochastic constraint (1.7), we have assumed the existence of a ‘back-to-labels’ map. This assumption is also often made in the derivation of the Navier–Stokes equations in a stochastic setting, e.g. [13,14]. For additional information, review and background references for random perturbations of PDEs and fluid dynamic models, viewed from complementary viewpoints to the present paper, see also Flandoli *et al.* [17,18]. In particular, Flandoli *et al.* [17,18] studies the interesting possibility that adding stochasticity can have a regularizing effect on fluid equations which might otherwise be ill-posed.

### (c) Plan of the paper

The remainder of the paper will derive the SPDEs for fluids in (1.9) and (1.10) from the variational principle *δS*=0 with stochastic action integral *S* given in (1.1), or equivalently (1.16). Towards this objective, we shall take the following steps.

Section 2 will derive the Stratonovich motion equations for *S* in (1.1) in the Lagrangian formulation. Section 3b will write the Itô representation of these equations. Section 4 will treat the abstract Kelvin circulation theorems in their Stratonovich 4a and Itô forms 4b. Section 5 will consider examples of stochastic fluid flows in the Stratonovich representation. Section 5a will discuss Stratonovich stochastic fluid flows without advected quantities. In particular, §5a will derive the Kelvin circulation theorem for Stratonovich stochastic fluids and verify their preservation of helicity (the linkage number for the vorticity field lines). Section 5b will derive the contributions of various advected quantities to the motion equation. Section 5c will treat the effects of these advected quantities in two examples of stochastic geophysical fluid dynamics (SGFD). These two stochastic GFD examples comprise the stochastic Euler–Boussinesq equations and the stochastic quasi-geostrophic (SQG) equations. In all of these examples, we will present both the Stratonovich and Itô forms of the stochastic fluid equations and contrast their implications. Section 6 will provide conclusions, further discussion and outlook.

We will proceed formally without addressing technical issues of stochastic analysis, by assuming all the objects we introduce in the paper are semimartingales. This assumption is possible because the parametric spatial dependence of the dynamical variables allows essentially finite-dimensional stochastic methods to be applied at each point of space. From that viewpoint, the paper presents a slight generalization of earlier work by Bismut [19], Lázaro-Camí & Ortega [20] and Bou-Rabee & Owhadi [21], which unifies their Hamiltonian and Lagrangian approaches to temporal stochastic dynamics, and extends them to the case of cylindrical noise in which spatial dependence is parametric, while temporal dependence is stochastic. Having made this assumption, we will be able to apply the normal rules of variational calculus to the Stratonovich integrals in Hamilton's principle (1.1) to derive the equations of motion. We will then transform the equations to the Itô side and derive the expected drift terms as generalizations of the second-order operators which first appeared in the original paper of Stratonovich [30]. A general principle will be given in theorem 2.1 and the results for a series of examples will be derived. The present approach introduces new stochastic terms into these examples which improve the geometric structure of the equations and preserve the invariants of the underlying deterministic models. These new stochastic terms contain multiplicative noise depending on the gradients of the solution variables. We hope this paper will stimulate new research, not only by experts in geometric mechanics, but also by those who approach stochastic fluid dynamics using more analytical methods, because the equations we derive here are new and pose new challenges.

## 2. Stratonovich stochastic variational principle

### (a) Stochastic Euler–Poincaré formulation

**Theorem 2.1 (Stratonovich Stochastic Euler–Poincaré equations)**

*The action for the SVP δS=0 in (1.16),*

2.1 $$S(u,p,q)=\underset{\mathrm{Lebesgue}\;\mathrm{integral}}{\underbrace{\int \left(\ell (u,q)+{\left\langle p,\frac{dq}{dt}+{£}_{u}q\right\rangle }_{V}\right)dt}}+\underset{\mathrm{Stratonovich}\;\mathrm{integral}}{\underbrace{\int \sum_{i}{\left\langle p⋄q,\;\xi_{i}(x)\right\rangle }_{X}\circ dW_{i}(t)}},$$

*leads to the following Stratonovich form of the Stochastic Euler–Poincaré (SEP) equations:*

2.2 $$dm+{£}_{dx_{t}}m-\frac{\delta \ell }{\delta q}⋄q\;dt=0,\;dq=-{£}_{dx_{t}}q\;\mathrm{and}\;dp=\frac{\delta \ell }{\delta q}\;dt+{£}_{dx_{t}}^{T}p,$$

*where* $dx_{t}\in X$ *is the Stratonovich stochastic vector field in equation (1.3) and*

2.3 $$m:=\frac{\delta \ell }{\delta u}=p⋄q\in X^{*}$$

*is the 1-form density of momentum.*

**Proof.**

The first step is to take the elementary variations of the action integral (2.1), to find

2.4 $$\delta u:\;\frac{\delta \ell }{\delta u}-p⋄q=0,\;\delta p:\;dq+{£}_{dx_{t}}q=0\;\mathrm{and}\;\delta q:\;\frac{\delta \ell }{\delta q}dt-dp+{£}_{dx_{t}}^{T}p=0.$$

The first variational equation captures the relation (2.3), and the latter two equations in (2.4) produce the corresponding equations in (2.2). The governing equation for *m* in (2.2) will be recovered by using the result of the following Lemma. ▪

**Lemma 2.2**

Together, the three equations in (2.4) imply the first formula in (2.2), namely

2.5 $$dm-\frac{\delta \ell }{\delta q}⋄q\;dt=-{£}_{dx_{t}}m.$$

**Proof.**

For an arbitrary η∈X, one computes the pairing

2.6 $$\begin{array}{rl} {\left\langle dm-\frac{\delta \ell }{\delta q}⋄q\;dt,\eta \right\rangle }_{X} & ={\left\langle -\frac{\delta \ell }{\delta q}⋄q+dp⋄q+p⋄dq,\eta \right\rangle }_{X}\\ \text{By equation (2.4)} & ={\left\langle ({£}_{dx_{t}}^{T}p)⋄q-p⋄{£}_{dx_{t}}q,\eta \right\rangle }_{X}\\ & ={\left\langle p,(-{£}_{dx_{t}}{£}_{\eta }+{£}_{\eta }{£}_{dx_{t}})q\right\rangle }_{V}\\ & ={\left\langle p,\;{\mathrm{ad}}_{dx_{t}}\eta q\right\rangle }_{V}=-{\left\langle p⋄q,\;{\mathrm{ad}}_{dx_{t}}\eta \right\rangle }_{X}\\ & =-{\left\langle {\mathrm{ad}}_{dx_{t}}^{*}(p⋄q),\eta \right\rangle }_{X}=-{\left\langle {£}_{dx_{t}}m,\eta \right\rangle }_{X}. \end{array}$$

Since η∈X was arbitrary, the last line completes the proof of the Lemma. In the last step, we have used the fact that coadjoint action is identical to Lie-derivative action for vector fields acting on 1-form densities.

In turn, the result of lemma 2.2 now produces the *m*-equation in (2.2) of theorem 2.1. ▪

## 3. Stratonovich → Itô equations

### (a) Stratonovich form

Since $dx_{t}=u\;dt-\sum_{i}\xi_{i}(x)\circ dW_{i}(t)$, one may rewrite the Stratonovich SEP equations (2.2) in theorem 2.1, so as to separate out the various Lie derivative operations, as follows:

3.1 $$\begin{array}{rl} dm+{£}_{u}m\;dt-\frac{\delta \ell }{\delta q}⋄q\;dt & =\sum_{i}{£}_{\xi_{i}(x)}m\circ dW_{i}(t),\\ dq+{£}_{u}q\;dt & =\sum_{i}{£}_{\xi_{i}(x)}q\circ dW_{i}(t)\\ \;\text{and}\;dp-{£}_{u}^{T}p\;dt-\frac{\delta \ell }{\delta q}\;dt & =-\sum_{i}{£}_{\xi_{i}(x)}^{T}p\circ dW_{i}(t), \end{array}\}$$

in which the stochastic process terms all appear on the right-hand sides of the equations. When those terms are absent, one recovers standard deterministic ideal fluid dynamics.

### (b) Itô form

The corresponding Itô forms of the equations in (3.1) are found by using Itô's formula to identify the quadratic covariation terms as

3.2 $$\begin{array}{rl} dm+{£}_{d{\hat{x}}_{t}}m\;dt-\frac{\delta \ell }{\delta q}⋄q\;dt & =\frac{1}{2}\sum_{j}{£}_{\xi_{j}(x)}({£}_{\xi_{j}(x)}m)\;dt,\\ dq+{£}_{d{\hat{x}}_{t}}q\;dt & =\frac{1}{2}\sum_{j}{£}_{\xi_{j}(x)}({£}_{\xi_{j}(x)}q)\;dt\\ \;\text{and}\;dp-{£}_{d{\hat{x}}_{t}}^{T}p\;dt-\frac{\delta \ell }{\delta q}\;dt & =-\frac{1}{2}\sum_{j}{£}_{\xi_{j}(x)}^{T}({£}_{\xi_{j}(x)}^{T}p)\;dt, \end{array}\}$$

where we have used [d*W*_*i*_(*t*), d*W*_*j*_]=*δ*_*ij*_ d*t* for Brownian motion and rearranged as shown in (3.8) below, in order to rewrite the Lie derivatives in terms of the stochastic Itô vector field, $d{\hat{x}}_{t}$, given in equation (1.11).

**Remark 3.1**

The right-hand sides in the Itô stochastic equations (3.2) define a second-order operator via double Lie derivatives with respect to the POD vector fields, *ξ*_*j*_(*x*), *j*=1,2,…,*K*. This seems like a type of the Laplace operator based on double Lie derivatives. To follow this intuition, we first introduce a *Lie-dual* *δ*_*ξ*_*j*__ to the exterior derivative ***d***, defined by the differential form operations

3.3 $$\delta_{\xi_{j}}q:=\xi_{j}\;⌟\;(\text{d}\;(\xi_{j}\;⌟\;q))\;\text{or, in other notation,}\;\delta_{\xi_{j}}q:=\iota_{\xi_{j}}\text{d}(\iota_{\xi_{j}}q),$$

where $\xi_{j}⌟$ and *ι*_*ξ*_*j*__ denote two standard expressions for the operation of insertion of a vector field into an arbitrary differential *k*-form *q*∈*Λ*^*k*^ and {*ξ*_*j*_} is taken as the given basis of POD vector fields associated with the spatial correlations of the cylindrical noise, enumerated in increasing order of eigenvalue by the subscript *j*. In terms of the Lie-dual operation *δ*_*ξ*_*j*__ and the exterior differential operator ***d***, we define the *Lie–Laplacian* operator Δ_Lie_ by

3.4 $$\sum_{j}{£}_{\xi_{j}}({£}_{\xi_{j}}q)=\sum_{j}(\delta_{\xi_{j}}\text{d}+\text{d}\delta_{\xi_{j}})q=:\Delta_{\mathrm{Lie}}q.$$

**Proposition 3.2**

*The Lie Laplacian operator* Δ_Lie_ *commutes with the exterior differential operator* ***d***. *That is*,

3.5 $$[\Delta_{\mathrm{Lie}},\text{d}]=\Delta_{\mathrm{Lie}}\text{d}-\text{d}\Delta_{\mathrm{Lie}}=0.$$

**Proof.**

This commutation relation follows immediately from the definition of the Lie Laplacian operator Δ_Lie_ in (3.4) and the property of the exterior differential that ***d***^2^*q*=0 when acting on a differentiable *k*-form *q*. It also follows from the definition (3.4) because Lie derivatives commute with exterior derivatives. ▪

**Remark 3.3 (Derivation of the Lie Laplacian operator in equation (3.2))**

Let us seek the Itô form of the stochastic part of the Stratonovich evolution in (3.1), ignoring drift. The stochastic part of the process in (3.1) can be written as a linear differential operator

3.6 $$dq(x,t)={£}_{\xi (x)}q(x,t)\circ dW(t),$$

in which we have ignored the drift term and suppressed indices on the vector fields *ξ*_*i*_(*x*) for simplicity of notation. Upon pairing this equation with a *time independent* test function *ϕ*(*x*)∈*V* *, we find the weak form of the Stratonovich advection equation (3.6),

3.7 $$d{\left\langle \phi (x),q\right\rangle }_{V}={\left\langle \phi (x),\;dq\right\rangle }_{V}={\left\langle \phi (x),{£}_{\xi (x)}q\circ dW(t)\right\rangle }_{V}={\left\langle {£}_{\xi (x)}^{T}\phi (x),q\right\rangle }_{V}\circ \;dW(t),$$

where we have taken advantage of the parametric *x*-dependence in *ϕ*(*x*) to pass the evolution operation *d* through it in the first step. Here, ${\left\langle \phi ,q\right\rangle }_{V}=\int <\phi (x),q(x,t)>dx$ denotes *L*^2^ integral pairing of a quantity *q*∈*V* with its tensor dual *ϕ*∈*V* * in the domain of flow, the symbol ${\text{ANAND }}_{\xi (x)}^{T}$ is the *L*^2^ adjoint of the Lie derivative £_*ξ*(*x*)_, and 〈⋅,⋅〉 denotes pairing between elements of *V* and their dual elements in *V* * at each point *x* in the domain. The corresponding weak form of the Itô evolution is

3.8 $$\begin{array}{rl} d{\left\langle \phi ,q\right\rangle }_{V} & ={\left\langle {£}_{\xi (x)}^{T}\phi ,q\right\rangle }_{V}\;dW(t)+\frac{1}{2}[d{\left\langle {£}_{\xi (x)}^{T}\phi ,q\right\rangle }_{V},\;dW(t)].\\ \text{By equation (3.7)} & ={\left\langle {£}_{\xi (x)}^{T}\phi ,q\right\rangle }_{V}\;dW(t)+\frac{1}{2}[{\left\langle {£}_{\xi (x)}^{T}({£}_{\xi (x)}^{T}\phi ),q\right\rangle }_{V}\;dW(t),\;dW(t)]\\ & ={\left\langle \phi ,{£}_{\xi (x)}q\right\rangle }_{V}\;dW(t)+\frac{1}{2}[{\left\langle \phi ,{£}_{\xi (x)}({£}_{\xi (x)}q)\right\rangle }_{V}\;dW(t),\;dW(t)]\\ & ={\left\langle \phi ,{£}_{\xi (x)}q\right\rangle }_{V}\;dW(t)+\frac{1}{2}{\left\langle \phi ,{£}_{\xi (x)}({£}_{\xi (x)}q)\right\rangle }_{V}\;dt, \end{array}$$

Hence, because *ϕ*(*x*)∈*V* * was chosen arbitrarily, the Itô form of the Stratonovich stochastic advection equation for *q*∈*V* in (3.1) is

3.9 $$dq+{£}_{u}q\;dt=\sum_{i}({£}_{\xi_{i}(x)}q)\;dW_{i}(t)+\frac{1}{2}\sum_{i,j}{£}_{\xi_{j}(x)}({£}_{\xi_{i}(x)}q)[dW{\left(t\right)}_{i},\;dW_{j}(t)].$$

For Brownian motion, the last term in (3.9) simplifies via [d*W*_*i*_(*t*), d*W*_*j*_]=*δ*_*ij*_ d*t*, and the middle equation in (3.2) emerges. Note that the last term in (3.9) (the quadratic Itô term) cannot be written as a Lie derivative of the Stratonovich-advected quantity *q*. Instead, it is a *double* Lie derivative, and this has the effect of masking the interpretation of the *q*-evolution in Itô form as advection.

## 4. Abstract Kelvin theorem

### (a) Stratonovich circulation theorem

Next, we shall define the ***circulation map*** $K:C\times V^{*}\to X{\left(D\right)}^{**}$, where C is a space of *material loops*, for which c∈C satisfies

4.1 $$dc(t)=-{£}_{dx_{t}}c(t).$$

Given a 1-form density $m\in X^{*}$ we can create a 1-form (no longer a density) by dividing it by the mass density, *D*. We denote the result just by *m*/*D*. We let the circulation map K then be defined by the integral of the 1-form *m*/*D* around a loop moving with the Stratonovich flow,

4.2 $$\langle K(c(t),q(t)),m\rangle =\oint_{c(t)}\frac{m}{D}.$$

The expression in this definition is called the ***circulation*** of the 1-form *m*/*D* around the loop *c*(*t*) moving along a Stratonovich stochastic path *x*_*t*_(*x*_0_) with *Eulerian* velocity vector field d*x*_*t*_ given as in equation (1.3),

4.3 $$dx_{t}=u(x,t)\;dt-\sum_{i}\xi_{i}(x)\circ \;dW_{i}(t).$$

Consider the stochastic flow equation for the loop integral $\oint_{c(t)}m/D$. The differential of this loop integral is the total differential of the integrand, as the loop *c*(*t*) itself is moving with the stochastic flow as in (4.1). Consequently, we find

4.4 $$d\oint_{c(t)}\frac{m}{D}=\oint_{c(t)}(d+{£}_{dx_{t}})\left(\frac{m}{D}\right)=\oint_{c(t)}\frac{1}{D}\frac{\delta \ell }{\delta q}⋄q\;dt,$$

where we have used equation (2.2) in theorem 2.1 as well as the advection relation for density *D*,

$$dD=-{£}_{dx_{t}}D=-{£}_{u(x,t)}D\;dt+\sum_{i}{£}_{\xi_{i}(x)}D\circ \;dW_{i}(t).$$

This calculation has proven the following theorem for the ***Kelvin–Noether circulation map*** $I:C\times V^{*}\times X\to R$ defined by:

4.5 $$I(t)=I(c,q,u):=\left\langle K(c,q),\frac{\delta l}{\delta u}(u,q)\right\rangle .$$

**Theorem 4.1 (Abstract Kelvin–Noether theorem for Stratonovich Euler–Poincaré SPDEs)**

*For* c(t)∈C, *let u(t),q(t) satisfy the reduced Stratonovich Euler–Poincaré SPDEs in (2.2) in theorem *2.1*. Choose the map* $K:C\times V^{*}\to X^{**}$ *given by integration around a loop c(t) satisfying (4.1). Then the Kelvin–Noether circulation map in (4.5) satisfies*

4.6 $$dI(t)=\left\langle K(t),\frac{1}{D}\frac{\delta l}{\delta q}⋄q\right\rangle dt.$$

**Remark 4.2**

Upon using the definition (4.2) for the Kelvin–Noether quantity in equation (4.5), one recovers the explicit formula (4.4) from the abstract Kelvin–Noether theorem in (4.6).

In particular, in the case of incompressible flow where *q*=*D*=1 (volume preservation) and *δl*/*δq*=0 for *q*≠*D*, then the Kelvin–Noether circulation map in (4.5) is *conserved*, as upon using the definitions of the circulation in (4.2) and the diamond operation in (1.15) we find,

4.7 $$d\oint_{c(t)}\frac{\delta \ell (u)}{\delta u}=\oint_{c(t)}(d+{£}_{dx_{t}})\frac{\delta \ell (u)}{\delta u}=0,$$

for any co-moving loop *c*(*t*), i.e. any loop satisfying equation (4.1), with stochastic vector field d*x*_*t*_ defined in (4.3).

### (b) Itô circulation theorem

As we have seen in equation (4.4), the Kelvin circulation theorem for the Stratonovich case is,

4.8 $$d\oint_{c(t)}\frac{1}{D}\frac{\delta \ell }{\delta u}=\oint_{c(t)}\frac{1}{D}\frac{\delta \ell }{\delta q}⋄q\;dt,$$

for loops *c*(*t*) satisfying equation (4.1) with the stochastic path velocity vector field d*x*_*t*_ defined in (4.3).

For the Itô case, according to equation (3.2) this circulation law becomes

4.9 $$d\oint_{\hat{c}(t)}\frac{1}{D}\frac{\delta \ell }{\delta u}=\oint_{\hat{c}(t)}\frac{1}{D}\frac{\delta \ell }{\delta q}⋄q+\oint_{\hat{c}(t)}\frac{1}{2}\sum_{i,j}{£}_{\xi_{j}(x)}\left({£}_{\xi_{i}(x)}\frac{1}{D}\frac{\delta \ell }{\delta u}\right)\delta_{ij}\;dt,$$

for loops $\hat{c}(t)$ following paths generated by flows of the Itô stochastic vector field $d{\hat{x}}_{t}$ given in equation (1.11).

Hence, the Itô equations have extra sources of circulation, even in the absence of advected quantities, *q*. In particular, the Kelvin–Noether result in theorem 4.1 shows that circulation is conserved for loops moving along the stochastic Stratonovich path when the advected quantities *q* are absent. However, not unexpectedly, because the velocities of the loops *c*(*t*) and $\hat{c}(t)$ are *different*, the Kelvin–Noether theorem shows that circulation is conserved *only* for loops moving along the stochastic Stratonovich path, *c*(*t*), and this conservation is *masked* in the Itô representation, because it does not hold for loops moving along the stochastic Itô path, $\hat{c}(t)$, and the quadratic covariation terms cannot be expressed as a single Lie derivative operation. Thus, as viewed along the Itô path, $\hat{c}(t)$, misalignment of the correlation eigenvectors creates or destroys circulation.

## 5. Examples

### (a) Stratonovich stochastic fluid flows without advected quantities

#### (i) Stratonovich stochastic Euler–Poincaré flows

The Stratonovich form of the *stochastic* Euler–Poincaré equations (2.2) in theorem 2.1 is, in the absence of advected quantities,

5.1 $$dv+{£}_{d{\text{x}}_{t}}v=-\text{d}p\;dt,$$

in which bold ***d*** denotes the *differential* in the standard exterior calculus sense, while italic *d* still denotes the stochastic change, including both the drift and the stochastic process. Here the variational derivative *v*=*δ*ℓ/*δu*∈*Λ*^1^ is the momentum 1-form dual to the velocity vector field. The divergence-free velocity vector field $u=\text{u}\cdot \nabla \in X(R^{3})$ in the covariant basis defined by the spatial gradient (∇) has vector components also written in bold as $\text{u}\in R^{3}$ which satisfy div ***u***=0. In fact, we shall assume that div ***ξ***_*i*_=0 with $\xi_{i}\in R^{3}$, also, so we may write the divergence of the stochastic path in equation (4.3) in vector form as

5.2 $$\mathrm{div}(d{\text{x}}_{t})=0,\;\text{with}\;d{\text{x}}_{t}=\text{u}(\text{x},t)\;dt-\sum_{i}\xi_{i}(\text{x})\circ dW_{i}(t).$$

This means the stochastic flow of the vector field *d****x***_*t*_ preserves volume elements. In this case, we may define the variational derivative 1-form density *δ*ℓ/*δu* in Eulerian spatial coordinates as simply the 1-form

$$v:=\frac{\delta \ell }{\delta u}=\text{v}\cdot \text{d}\text{x}.$$

The Lie derivative in equation (5.1) is then written in vector form, so that equation (5.1) becomes

$$(d\text{v})\cdot \text{d}\text{x}+{£}_{dx_{t}}(\text{v}\cdot \text{d}\text{x})=(d\text{v}+d{\text{x}}_{t}\cdot \nabla \text{v}+{\left(\nabla d{\text{x}}_{t}\right)}^{T}\cdot \text{v})\cdot \text{d}\text{x}=-\nabla p\cdot \text{d}\text{x}\;dt.$$

In three-dimensional vector form, this equation is

5.3 $$\begin{array}{rl} d\text{v}+d{\text{x}}_{t}\cdot \nabla \text{v}+{\left(\nabla d{\text{x}}_{t}\right)}^{T}\cdot \text{v} & =-\nabla p\;dt\\ & =d\text{v}-d{\text{x}}_{t}\times \mathrm{curl}\;\text{v}+\nabla (d{\text{x}}_{t}\cdot \text{v}). \end{array}$$

Taking the curl of equation (5.3) yields the stochastic equation for the vorticity ***ω***=curl ***v***

5.4 $$d\omega -\mathrm{curl}(d{\text{x}}_{t}\times \omega )=0.$$

We define the *vorticity flux* as a 2-form *ω* with basis area element ***dS*** as

5.5 ω:=dv=d(v⋅dx)=(curl v)⋅dS=ω⋅dS.

Consequently, we may write the vorticity vector equation (5.4) in geometric form as

5.6 $$d\omega +{£}_{dx_{t}}\omega =0.$$

This equation also follows directly from the exterior derivative of the geometric form of the motion equation in (5.1) by invoking commutation of the Lie derivative and the exterior derivative. It means the flux of vorticity ***ω***⋅***dS***_*t*_ through a surface element following the stochastic path ***x***=***x***_*t*_(***x***_0_) is invariant. For an alternative formulation of the stochastic fluid vorticity equations in terms of a nonlinear version of the Feynman–Kac formula, see [31].

**Remark 5.1 (Itô stochastic Euler–Poincaré equations)**

The Itô forms of the motion equation in (5.1) and vorticity equation (5.6) for incompressible motion in the absence of advected quantities are given by

5.7 $$\begin{array}{rl} dv+{£}_{d{\hat{x}}_{t}}v & =-\text{d}p+\frac{1}{2}\sum_{i,j}{£}_{\xi_{j}(x)}({£}_{\xi_{i}(x)}v)\delta_{ij}\;dt\\ \;\text{and}\;\;d\omega +{£}_{d{\hat{x}}_{t}}\omega & =\frac{1}{2}\sum_{i,j}({£}_{\xi_{j}(x)}({£}_{\xi_{i}(x)}\omega ))\delta_{ij}\;dt, \end{array}\}$$

where the vorticity 2-form *ω*=***ω***⋅***dS*** is given in (5.5), and we have used commutation of exterior derivative ***d*** and Lie derivative £_*ξ*_*j*_(*x*)_ twice.

#### (ii) Helicity preservation for Stratonovich stochastic Euler–Poincaré flows

**Definition 5.2 (Helicity)**

The helicity *Λ*[curl ***v***] of a divergence-free vector field curl ***v*** that is tangent to the boundary ∂*D* of a simply connected domain $D\in R^{3}$ is defined as

5.8 $$\Lambda [\mathrm{curl}\;\text{v}]=\int_{D}(\text{v}\cdot \mathrm{curl}\;\text{v}){\text{d}}^{3}x,$$

where ***v*** is a divergence-free vector-potential for the field curl ***v*** and ***d***^3^*x* is the spatial volume element.

**Remark 5.3**

The helicity of a vector field curl ***v*** measures the average linking of its field lines, or their relative winding. For excellent historical surveys refer to Arnold & Khesin [32] and Moffatt & Tsinober [33] . The helicity is unchanged by adding a gradient to the vector ***v***, and div ***v***=0 is not a restriction for simply connected domains in $R^{3}$, provided curl ***v*** is tangent to the boundary ∂*D*.

The principal feature of this concept for Stochastic Euler flows is embodied in the following theorem.

**Theorem 5.4 (Stratonovich stochastic Euler flows preserve helicity)**

*When homogeneous or periodic boundary conditions are imposed, Euler's equations for an ideal incompressible fluid flow preserves the helicity, defined as the volume integral*

5.9 $$\Lambda [\mathrm{curl}\;\text{v}]=\int_{D}\text{v}\cdot \mathrm{curl}\;\text{v}d^{3}x=\int_{D}v\wedge \text{d}v,$$

*where v=δℓ/δu=****v****⋅****dx*** *is the circulation 1-form,* ***d****v=curl ****v****⋅****dS*** *is the vorticity flux (a 2-form), curl ****v****=****ω*** *is the vorticity vector and d*^3^*x is the spatial volume element.*

**Proof.**

Rewrite the geometric form of the Stochastic Euler equations (5.1) for rotating incompressible flow with unit mass density in terms of the circulation 1-form *v*:=***v***⋅***dx*** as

5.10 $$dv+{£}_{dx_{t}}v=-\text{d}p\;dt.$$

and £_*dx*_*t*__***d***^3^*x*=div(*d****x***_*t*_)*d*^3^*x*=0. Then the ***helicity density***, defined as

5.11 $$v\wedge \text{d}v=(\text{v}\cdot \mathrm{curl}\;\text{v})d^{3}x=\lambda d^{3}x,\;\text{with}\;\lambda =\text{v}\cdot \mathrm{curl}\;\text{v},$$

obeys the dynamics it inherits from the Stochastic Euler equations,

5.12 $$(d+{£}_{dx_{t}})(v\wedge \text{d}v)=-(\text{d}p\wedge \text{d}v)\;dt-(v\wedge {\text{d}}^{2}p)\;dt=-(\text{d}(p\;\text{d}v))\;dt,$$

after using ***d***^2^*p*=0 and ***d***^2^*v*=0. In vector form, this result may be expressed as a conservation law,

5.13 $$(d\lambda +\mathrm{div}\;\lambda \;d{\text{x}}_{t})\;{\text{d}}^{3}x=-\mathrm{div}(p\;curl\;\text{v}){\text{d}}^{3}x\;dt.$$

Consequently, the time derivative of the integrated helicity in a domain *D* obeys

5.14 $$\begin{array}{rl} d\Lambda [\mathrm{curl}\;\text{v}] & =\int_{D}(d\lambda ){\text{d}}^{3}x=-\int_{D}\mathrm{div}(\lambda \;d{\text{x}}_{t}+p\;curl\;\text{v}\;dt){\text{d}}^{3}x\\ & =-\oint_{\partial D}(\lambda \;d{\text{x}}_{t}+p\;curl\;\text{v}\;dt)\cdot \hat{\text{n}}\;\text{d}S, \end{array}$$

which vanishes when homogeneous or periodic boundary conditions are imposed on ∂*D*. ▪

**Corollary 5.5 (The Itô representation of stochastic Euler flows masks the Stratonovich preservation of helicity)**

**Proof.**

Equation (5.7) for Itô Euler–Poincaré incompressible flow yields, for *ω*=***d****v* as before,

5.15 $$\begin{array}{rl} \left(\partial_{t}+{£}_{d{\hat{x}}_{t}/dt})(v\wedge \omega \right) & =(-\text{d}p+\frac{1}{2}\Delta_{\mathrm{Lie}}v)\wedge \omega +v\wedge \frac{1}{2}\Delta_{\mathrm{Lie}}\omega \\ & =-\text{d}(p\omega +v\wedge \frac{1}{2}\Delta_{\mathrm{Lie}}v)+v\wedge \Delta_{\mathrm{Lie}}\omega . \end{array}$$

In vector form, the last equation in (5.15) may be integrated over space and written as

5.16 $$\frac{d}{dt}\int_{D}(\text{v}\cdot \omega ){\text{d}}^{3}x=-\int_{D}\mathrm{div}\left((\text{v}\cdot \omega )\frac{d{\hat{\text{x}}}_{t}}{dt}+p\omega +\frac{1}{2}\text{v}\times \Delta_{\mathrm{Lie}}\omega \right){\text{d}}^{3}x+\int_{D}(\text{v}\cdot \Delta_{\mathrm{Lie}}\omega ){\text{d}}^{3}x.$$

 ▪

**Remark 5.6**

Hence, even for homogeneous or periodic boundary conditions, in which the integral of the divergence would vanish, there remains a non-vanishing term on the right-hand side of equation (5.16) for the Itô evolution of the helicity, due to the additional quadratic drift term arising in the Itô calculus. Thus, the Itô stochastic dynamics appears to predict reconnection of vorticity field lines, although their linkages are actually *preserved* in the Stratonovich representation. Hence, as usual, one must take caution in drawing conclusions about stochastic fluid dynamics, because some of its features may be representation-dependent.

### (b) The effects of advected quantities

In this section, we compute the explicit formulae needed in applications of the system of stochastic equations in (2.2) for the vector space *V* of three-dimensional quantities *q*∈*V* consisting of elements with the following coordinate functions in three-dimensional Euclidean vector notation,

5.17 $$q\in \{b,\text{A}\cdot \text{d}\text{x},\text{B}\cdot \text{d}\text{S},D{\text{d}}^{3}x\}=:V.$$

Dual quantities under the *L*^2^ pairing are (*b*,*Dd*^3^*x*) and (**A**⋅***dx***,**B**⋅***dS***). The vector space *V* contains the geometric quantities that typically occur in ideal continuum dynamics. These are scalar functions (*b*), 1-forms (**A**⋅***dx***), 2-forms (**B**⋅***dS***) and densities (*Dd*^3^*x*) in three dimensions. In addition, with applications to magnetohydrodynamics (MHD) in mind, we also choose **B**=curl **A** and ***d***(**A**⋅***dx***)=**B**⋅***dS***. In Euclidean coordinates on $R^{3}$, this is $\text{d}(A_{k}\;\text{d}x^{k})=A_{k,j}\;\text{d}x^{j}\wedge \text{d}x^{k}=\frac{1}{2}\epsilon_{ijk}B^{i}\;\text{d}x^{j}\wedge \text{d}x^{k}$, where *ϵ*_*ijk*_ is the completely antisymmetric tensor density on $R^{3}$ with *ϵ*_123_=+1. The 2-form **B**⋅***dS***=***d***(**A**⋅***dx***) is the physically interesting special case of *B*_*kj*_ *dx*^*j*^∧ *dx*^*k*^ for MHD, in which *B*_*kj*_=*A*_*k*,*j*_, so that ∇⋅**B**=0.

**Definition 5.7 (Deterministic advection relations for *q*∈*V* in (5.17))**

The deterministic advection relations ∂_*t*_*q*=−£_*u*_*q* for the quantities *q*∈*V* in (5.17) are given explicitly by the Lie-derivative action of smooth vector fields $u\in X(R^{3})$ on the vector space of variables *q*∈*V* . These deterministic advection relations are given by

5.18 $$\begin{array}{rl} \partial_{t}b & =-{£}_{u}b=-\text{u}\cdot \nabla b,\\ \partial_{t}\text{A}\cdot \text{d}\text{x} & =-{£}_{u}(\text{A}\cdot \text{d}\text{x})=-((\text{u}\cdot \nabla )\text{A}+A_{j}\nabla u^{j})\cdot \text{d}\text{x}\\ & =(\text{u}\times \mathrm{curl}\text{A}-\nabla (\text{u}\cdot \text{A}))\cdot \text{d}\text{x},\\ \partial_{t}\text{B}\cdot \text{d}\text{S} & =-{£}_{u}(\text{B}\cdot \text{d}\text{S})=(\mathrm{curl}\;(\text{u}\times \text{B}))\cdot \text{d}\text{S}=d(\partial_{t}\text{A}\cdot \text{d}\text{x})\\ \;\text{and}\;\partial_{t}D\;{\text{d}}^{3}x & =-{£}_{u}(D{\text{d}}^{3}x)=-\nabla \cdot (D\text{u}){\text{d}}^{3}x. \end{array}\}$$

*The diamond operation.* The diamond operation $(⋄):T^{*}V\to X^{*}$ is defined for (*q*,*p*)∈T*V and $u\in X(R^{3})$ by equation (1.15) as

5.19 $${\left\langle p⋄q,u\right\rangle }_{X}={\left\langle p,-{£}_{u}q\right\rangle }_{V},$$

for the *L*^2^ pairings ${\left\langle \cdot ,\cdot \right\rangle }_{V}:T^{*}V\times TV\to R$ and ${\left\langle \cdot ,\cdot \right\rangle }_{X}:X^{*}\times X\to R$ with $p⋄q\in X^{*}$. Under the *L*^2^ pairing, we assume that boundary terms arising from integrations by parts may be dropped, by invoking natural boundary conditions.

In particular, for the set of advected quantities *q*∈*V* in (5.17) above, we find the following Euclidean components of the sum of terms δℓ/δq⋄q in the motion equation (2.2), for stochastic EP equations,

5.20 $${\left(\frac{\delta \ell }{\delta q}⋄q\right)}_{k}=-\frac{\delta \ell }{\delta b}{\left(\nabla b\right)}_{k}+D{\left(\nabla \frac{\delta \ell }{\delta D}\right)}_{k}+{\left(-\frac{\delta \ell }{\delta \text{A}}\times \mathrm{curl}\;\text{A}+\text{A}\;div\;\frac{\delta \ell }{\delta \text{A}}+\text{B}\times \mathrm{curl}\;\frac{\delta \ell }{\delta \text{B}}\right)}_{k}.$$

With these definitions, one may write explicit formulae for the fluid examples needed in applications of the system of stochastic equations in (2.2) for deterministic advected quantities *q* in the vector space *V* of three-dimensional quantities in (5.17). These applications include, for example, GFD and magnetohydrodynamics (MHD). The applications of the present theory to MHD will be pursued elsewhere. In this paper, we restrict ourselves to examples from GFD.

### (c) Stochastic geophysical fluid dynamics

#### (i) Euler–Boussinesq approximation

*Stochastic Euler–Boussinesq equations of a rotating stratified incompressible fluid.* In SGFD, the SEP equations in (2.2) are found in the Euler–Boussinesq approximation, for example, by choosing the Lagrangian ℓ(*u*,*b*,*D*) depending on the set *q*∈{*b*,*D*} and given by [34]

5.21 $$\ell (u,b,D)=\int \frac{D}{2}|u{|}^{2}+Du\cdot R-gbDz-p(D-1){\text{d}}^{3}x,$$

where *u* is fluid velocity, *D* is the volume element, 2*Ω*=curl*R*(*x*) is the Coriolis vector, while *R*(*x*), a given function of *x* is its vector potential, *g* is the constant gravitational acceleration, *b* is buoyancy and the pressure *p* is a Lagrange multiplier which enforces *D*=1, so that div *u*=0. The GFD Lagrangian (5.21) possesses the following variations at fixed *x* and *t*,

5.22 $$\begin{array}{rl} \frac{m}{D} & =\frac{1}{D}\frac{\delta l}{\delta u}=u+R(x),\;\frac{\delta l}{\delta b}=-Dgz\\ \;\mathrm{and}\;\frac{\delta l}{\delta D} & =\frac{1}{2}|u{|}^{2}+u\cdot R-gzb-p,\;\frac{\delta l}{\delta p}=-(D-1). \end{array}\}$$

Hence, from the SEP equations in (2.2), we find the motion equation for an Euler–Boussinesq fluid in three dimensions,

5.23 $$du+{£}_{dx_{t}}(u+R(x))=-gb\nabla z\;dt+\nabla (-p+\frac{1}{2}|u{|}^{2}+u\cdot R)\;dt\;\text{and}\;db+{£}_{dx_{t}}b=0,$$

with *dx*_*t*_ given as before in (1.3) and (4.3).^3^ Consequently, we find the stochastic advection law,

5.24 $$(d+{£}_{dx_{t}})Q=dQ+d{\text{x}}_{t}\cdot \nabla Q=0,$$

for the potential vorticity, *Q*, defined by its traditional formula,

5.25 Q:=curl(u+R(x))⋅∇b,

where the total vorticity *ω*=curl(*u*+*R*(*x*)) satisfies

5.26 $$d\omega =\mathrm{curl}(dx_{t}\times \omega )-g\nabla b\times \nabla z\;dt,\;\text{with}\;db+dx_{t}\cdot \nabla b=0.$$

These results are implied by the Stratonovich circulation theorem in §4a. The stochastic conservation law for the potential vorticity *Q* in equation (5.24), means that *Q* is preserved along *each* Stratonovich stochastic path.

*Itô form of the potential vorticity equation.* The interpretation of the Itô form of the potential vorticity equation (5.24) may be obtained by expanding it out using the Itô equations in (3.2). Indeed, the Itô form of the Stratonovich equation (5.24) for potential vorticity *Q* is the precisely the same as the double Lie-derivative formula for the advected quantity *q* in equation (3.2); namely,

5.27 $$dQ+{£}_{d{\hat{x}}_{t}}Q\;dt=\frac{1}{2}\sum_{i,j}{£}_{\xi_{j}(x)}({£}_{\xi_{i}(x)}Q)\delta_{ij}\;dt,$$

or, in vector calculus form,

5.28 $$dQ+d{\hat{\text{x}}}_{t}\cdot \nabla Q=\frac{1}{2}\sum_{i,j}\xi_{j}(\text{x})\cdot \nabla (\xi_{i}(\text{x})\cdot \nabla Q)\delta_{ij}\;dt.$$

Upon comparing equations (5.24) and (5.28), one concludes that the stochastic Euler–Boussinesq equations (5.23) in three dimensions preserve the potential vorticity *Q* defined in equation (5.25) along the Stratonovich stochastic path *x*_*t*_(*x*) but *Q* preservation is *masked* in the Itô representation, because *Q* is not preserved along the Itô stochastic path ${\hat{\text{x}}}_{t}(x)$, which of course is a *different* path. The operator Δ_Lie_ in the last term in the Itô equation (5.28) reduces to the metric Laplacian Δ=∇^2^ in the case that the vectors ***ξ***_*j*_ with *j*=1,2,3, are linearly independent unit vectors in three dimensions.

#### (ii) Quasi-geostrophic approximation

*Deterministic quasi-geostrophic equations.* The quasi-geostrophic (QG) approximation is a fundamental model which is often used for the analysis of meso- and large-scale motion in geophysical and astrophysical fluid dynamics [35]. Physically, the QG approximation applies when the motion is nearly in geostrophic balance, i.e. when pressure gradients nearly balance the Coriolis force. In the simplest case of a barotropic fluid in a domain D on the plane $R^{2}$ with coordinates (*x*_1_,*x*_2_), geostrophic balance determines the geostrophic fluid velocity ***u*** as $\text{u}:=\hat{\text{z}}\times \nabla \psi ,$ where *ψ* is the stream-function, the flow is incompressible (∇⋅***u***=0) and $\hat{\text{z}}$ is the unit vector normal to the plane.

The QG dynamics in the *β*-plane approximation is expressed by the following evolution equation for the stream-function *ψ* of the geostrophic fluid velocity ***u***,

5.29 $$\frac{\partial (\Delta \psi -F\psi )}{\partial t}+{\left[\psi ,\Delta \psi \right]}_{\mathrm{Jac}}+\beta \frac{\partial \psi }{\partial x_{1}}=0,$$

where ∂/∂*t* is the partial time derivative, Δ is the planar Laplacian, F denotes rotational Froude number, the square bracket [⋅,⋅]_Jac_ denotes

5.30 $${\left[a,b\right]}_{\mathrm{Jac}}:=\frac{\partial (a,b)}{\partial (x_{1},x_{2})}=\hat{\text{z}}\cdot \nabla a\times \nabla b,$$

which is the Jacobi bracket (Jacobian) for functions *a* and *b* on $R^{2}$, and *β* is the *x*_2_-gradient of the Coriolis parameter, *f*, taken as *f*=*f*_0_+*βx*_2_ in the *β*-plane approximation, with constants *β* and *f*_0_. Neglecting *β* gives the *f*-plane approximation.

The QG equation (5.29) may be derived from the basic equations of rotating shallow water flow by rescaling to define non-dimensional variables and making an asymptotic expansion in terms of the Froude number given by the square of the ratio of the characteristic scale of the motion to the deformation radius, e.g. Pedlosky [35], Allen & Holm [36] and Zeitlin & Pasmenter [37], for details of the QG derivation.

Equation (5.29) may be written alternatively as an advection law for the *potential vorticity*, *Q*,

5.31 $$\frac{\partial Q}{\partial t}=-\text{u}\cdot \nabla Q=-\hat{\text{z}}\times \nabla \psi \cdot \nabla Q,\;\text{where}\;Q:=\Delta \psi -F\psi +f.$$

This form of QG dynamics emphasizes its basic property; namely, potential vorticity *Q* is conserved on geostrophic fluid parcels.

Much is known about the mathematical structure of the deterministic QG equations (5.29) and (5.31). In particular, they are Hamiltonian with a Lie–Poisson bracket given in Weinstein [38] as

5.32 $$\{F,H\}=-\int Q{\left[\frac{\delta F}{\delta \mu },\frac{\delta H}{\delta \mu }\right]}_{\mathrm{Jac}}\;dx_{1}\wedge dx_{2},$$

where *μ*:=*Q*−*f* and the square bracket [⋅,⋅]_Jac_ is the Jacobi bracket in (5.30). In terms of the variable *μ*, the Hamiltonian for QG is expressed as

5.33 $$H=\frac{1}{2}\int_{D}(|\nabla \psi {|}^{2}+F\psi^{2})\;\text{d}x_{1}\wedge \text{d}x_{2}=\frac{1}{2}\int_{D}\mu {\left(F-\Delta \right)}^{-1}\mu \;\text{d}x_{1}\wedge \text{d}x_{2}+\frac{1}{2}\sum_{i}\oint_{\gamma_{i}}\psi \text{u}\cdot \text{d}\text{x},$$

where *γ*_*i*_ is the *i*th connected component of the boundary ∂D. In what follows, we will discuss cases where the domain D is either a torus (periodic boundary conditions) or the whole plane $R^{2}$ with decaying boundary conditions and, thus, the boundary terms may be ignored. Hence, we may write the Hamiltonian in terms of the *L*^2^ pairing 〈⋅,⋅〉_*L*^2^_ as

5.34 $$H(\mu )=\frac{1}{2}{\left\langle \mu ,{\left(F-\Delta \right)}^{-1}\mu \right\rangle }_{L^{2}}\;\text{with}\;\frac{\delta H}{\delta \mu }={\left(F-\Delta \right)}^{-1}\mu =\psi .$$

Consequently, the Lie–Poisson bracket (5.32) gives, after integration by parts, the dynamical equation for *μ*,

5.35 $$\frac{\partial \mu }{\partial t}=\{\mu ,H\}=-{\left[\psi ,Q\right]}_{\mathrm{Jac}}=-\text{u}\cdot \nabla Q,$$

in agreement with the QG potential vorticity equation (5.31). Casimirs of the Lie–Poisson bracket (5.32) are given by

5.36 $$C_{\Phi }=\int \Phi (Q)\;\text{d}x_{1}\wedge \text{d}x_{2},$$

for an arbitrary function *Φ* and they satisfy {*C*_*Φ*_,*H*}=0 for all Hamiltonians *H*(*μ*). Level surfaces of the Casimirs *C*_*Φ*_ define coadjoint orbits of the group of symplectic diffeomorphisms of the domain of the flow [38].

*Stochastic quasi-geostrophic equations.* The SQG may be derived by applying the Lie–Poisson structure in (5.32) to a stochastic Hamiltonian. Thus, to introduce stochastic forcing into the QG equations, we propose to augment the QG Hamiltonian in (5.34) with a Stratonovich stochastic term, as

5.37 $$h\;dt={\left\langle \mu ,\frac{1}{2}{\left(F-\Delta \right)}^{-1}\mu \;dt-\sum_{i}\xi_{i}(\text{x})\circ dW_{i}\right\rangle }_{L^{2}},$$

in which the {*ξ*_*i*_(***x***)} are prescribed spatial functions. The variational derivative of the augmented QG Hamiltonian in (5.37) yields the Stratonovich *stochastic stream function*

5.38 $$\frac{\delta h}{\delta \mu }\;dt=\psi (\text{x},t)\;dt-\sum_{i}\xi_{i}(\text{x})\circ dW_{i}=:\Psi \;dt.$$

Consequently, the Lie–Poisson bracket (5.32) gives, after integration by parts, the dynamical equation for *μ*,

5.39 $$d\mu =\{\mu ,h\;dt\}=-{\left[\Psi \;dt,Q\right]}_{\mathrm{Jac}}=-{\left[\psi ,Q\right]}_{\mathrm{Jac}}\;dt+\sum_{i}{\left[\xi_{i}(\text{x}),Q\right]}_{\mathrm{Jac}}\circ dW_{i}=-\text{d}{\text{x}}_{t}\cdot \nabla Q,$$

in which, cf. equation (4.3),

5.40 $$d{\text{x}}_{t}:=\hat{\text{z}}\times \nabla \Psi \;dt=\hat{\text{z}}\times \nabla \left(\psi \;dt-\sum_{i}\xi_{i}(\text{x})\circ dW_{i}\right)=\text{u}\;dt-\sum_{i}(\hat{\text{z}}\times \nabla \xi_{i}(\text{x}))\circ dW_{i}.$$

This result is in agreement with the advection law in the stochastic equation (5.24) for potential vorticity in the Euler–Boussinesq approximation, provided we identify $\hat{\text{z}}\times \nabla \xi_{i}(\text{x})$ for these stochastic QG equations with the $R^{3}$ vector functions ***ξ***_*i*_(***x***) for the stochastic Euler–Bouusinesq equations in the previous example.

Of course, the Casimirs of the Lie–Poisson bracket (5.32) given by $C_{\Phi }=\int \Phi (Q)\;\text{d}x_{1}\wedge \text{d}x_{2}$ for an arbitrary function *Φ* are still conserved along the Stratonovich stochastic path, since this property holds for the Lie–Poisson bracket, independently of the choice of a stochastic Hamiltonian.

*Itô form.* The Itô form of equation (5.39) is,

5.41 $$d\mu =-{\left[\psi \;dt-\sum_{i}\xi_{i}(\text{x})\;dW_{i},Q\right]}_{\mathrm{Jac}}+\frac{1}{2}\sum_{j}{\left[\xi_{j}(\text{x}),{\left[\xi_{j}(\text{x}),Q\right]}_{\mathrm{Jac}}\right]}_{\mathrm{Jac}}\;dt,$$

where we have assumed that the Itô stochastic processes *dW*_*i*_(*t*) and *dW*_*j*_(*t*) are Brownian processes, whose quadratic covariations satisfy [*dW*_*i*_(*t*),*dW*_*j*_(*t*)]=*δ*_*ij*_ *dt* [16]. This SQG version of the Itô dynamics for potential vorticity *Q* is reminiscent of potential vorticity dynamics for dissipative QG with viscosity.

## 6. Conclusion

The stochastically constrained variational principle *δS*=0 for the action *S* given in (1.1) for introducing Stratonovich stochasticity into Euler–Poincaré equations for continuum dynamics has yielded stochastic incompressible flows that were found to preserve three important properties of ideal incompressible Euler flows which arise from its invariance under relabelling of Lagrangian coordinates. These three properties are: (i) the Kelvin circulation theorem in equation (4.7), (ii) invariance of the flux of vorticity in (5.6) through any surface element following the Stratonovich stochastic path *x*_*t*_ in (4.3), and (iii) preservation of the linkage number for the vorticity field lines (helicity) in theorem 5.4. The Kelvin circulation theorem is preserved because the material fluid loops follow the Stratonovich stochastic paths, as do the field lines of the vorticity, so all three results follow from the same interpretation. Likewise, the stochastic conservation law for the potential vorticity *Q*, found in equation (5.24) for the Euler–Boussinesq equations with Stratonovich noise, meant that *Q* was preserved along *each* Stratonovich stochastic path. The same type of potential vorticity preservation along Stratonovich stochastic paths was found again for the case of SQG in §5c(ii).

By contrast, the Itô representation of the stochastic equations involved a different stochastic vector field $d{\hat{x}}_{t}$ in (1.11) whose drift velocity contained the additional quadratic term that arises in Itô calculus. This quadratic Itô drift velocity introduced terms that were not present in the Stratonovich stochastic equations and could not be expressed as single Lie derivatives. As a result, the Itô representation masked the preservation of ideal incompressible Euler flow properties and conservation of potential vorticity which was found to hold in the Stratonovich case.

The Itô drift term turned out to contain an interesting Laplacian-like operator, Δ_Lie_, defined in equation (3.4) by a sum over double Lie derivatives with respect to the POD vector fields, *ξ*_*j*_(*x*), *j*=1,2,…,*K*, as $\Delta_{\mathrm{Lie}}:=\sum_{j}{\text{ANAND }}_{\xi_{j}(x)}({\text{ANAND }}_{\xi_{j}(x)}\cdot )$, which we called the *Lie Laplacian* operator, since £_*ξ*_*j*_(*x*)_ denotes the Lie derivative with respect to the vector field *ξ*_*j*_(*x*). This term is the generalization for advected quantities *q*∈*V* , in an arbitrary vector space *V* , of the quadratic covariation drift term found already for scalar densities by Stratonovich [30]. The Lie Laplacian operator appearing, for example, in the Itô representation of the stochastic fluid equations (3.2) is not a standard Laplacian operator, although it reduces to the metric Laplacian when the independent vector fields *ξ*_*j*_(*x*) for stochastic spatial correlations are constant and *j*=1,2,3. It would be interesting to know whether (because of its relation to the metric Laplacian) the Lie Laplacian operator in the Itô representation could have a regularizing effect on stochastic fluid equations which might otherwise be ill-posed, as suggested in Flandoli *et al.* [17,18].

## Footnotes

1 PODs are also called empirical orthogonal functions (EOFs), Karhunen–Loeve projections (KLPs) and singular value decompositions (SVDs), and they comprise a standard approach that has been useful in a variety of contexts [3].

2 The symbol *ω* for stochastic quantities will be understood throughout, but will not be written explicitly hereafter.

3 In this section, we distinguish between a vector field $dx_{t}\in X(R^{3})$ and its vector representation in a Cartesian basis, $d{\text{x}}_{t}\in R^{3}$. For example, with this notation one may write *dx*_*t*_=*d****x***_*t*_⋅∇.
